# Event-Triggered Secure Control Design Against False Data Injection Attacks via Lyapunov-Based Neural Networks

**DOI:** 10.3390/s25123634

**Published:** 2025-06-10

**Authors:** Neslihan Karas Kutlucan, Levent Ucun, Janset Dasdemir

**Affiliations:** 1 Control and Automation Engineering, Yildiz Technical University, 34220 Istanbul, Türkiye; lucun@yildiz.edu.tr; 2 Control and Mechatronics, University of Twente, P.O. Box 217, 7500 AE Enschede, The Netherlands; j.dasdemir@utwente.nl

**Keywords:** cyber-physical systems, event-triggered control, false data injection attack, linear matrix inequality, neural network, secure control

## Abstract

This paper presents a secure control framework enhanced with an event-triggered mechanism to ensure resilient and resource-efficient operation under false data injection (FDI) attacks on sensor measurements. The proposed method integrates a Kalman filter and a neural network (NN) to construct a hybrid observer capable of detecting and compensating for malicious anomalies in sensor measurements in real time. Lyapunov-based update laws are developed for the neural network weights to ensure closed-loop system stability. To efficiently manage system resources and minimize unnecessary control actions, an event-triggered control (ETC) strategy is incorporated, updating the control input only when a predefined triggering condition is violated. A Lyapunov-based stability analysis is conducted, and linear matrix inequality (LMI) conditions are formulated to guarantee the boundedness of estimation and system errors, as well as to determine the triggering threshold used in the event-triggered mechanism. Simulation studies on a two-degree-of-freedom (2-DOF) robot manipulator validate the effectiveness of the proposed scheme in mitigating various FDI attack scenarios while reducing control redundancy and computational overhead. The results demonstrate the framework’s suitability for secure and resource-aware control in safety-critical applications.

## 1. Introduction

Autonomous intelligent systems, encompassing technologies such as industrial automation, autonomous vehicles, and advanced robotics, are transforming modern industries by enabling complex and high-precision operations in safety-critical environments. Among these, robot manipulators are frequently employed in domains such as industrial manufacturing [1], medical rehabilitation [2], space exploration [3], and agricultural automation [4], where precision, reliability, and autonomy are essential. Equipped with various sensors and advanced control algorithms, robot manipulators can dynamically respond to environmental changes and optimize task execution, as demonstrated in [5], significantly enhancing both operational efficiency and safety in hazardous or inaccessible environments. However, the occurrence of cyber-attacks remains a critical threat, as such attacks can compromise both the performance and safety of robot manipulators [6].

Cyber-attacks may distort sensor readings, leading to incorrect environmental interpretation, unpredictable behaviors, and potential collisions. For instance, a false data injection (FDI) attack targeting a position sensor can cause the manipulator to deviate from its intended trajectory, resulting in serious reliability degradation [7]. This threat becomes even more critical in human–robot interaction (HRI) scenarios, where the physical proximity between humans and robots increases the risk. Malicious data manipulation under FDI attacks can lead to hazardous interactions, unpredictable robotic behavior, and failures in cooperative tasks. Therefore, real-time detection and mitigation of such threats are essential to ensure safe, trustworthy, and human-centric robotic assistance in both industrial and service environments. Moreover, the implications of FDI attacks are not limited to robotic manipulators; similar risks extend across a wide range of cyber–physical systems [8], where compromised sensor data may lead to unsafe control decisions and system responses in safety-critical operations.

In response to increasing cyber-attack threats, secure control strategies have been developed to maintain system performance in the presence of cyber-attacks [9]. These strategies enable systems to compensate for detected anomalies or malicious data manipulations, ensuring continuous and accurate operation even under adversarial scenarios. A crucial aspect of such frameworks is the detection of sensor anomalies, which is typically addressed through model-based, learning-based, and hybrid approaches. Model-based methods rely on the mathematical modeling of system dynamics to detect inconsistencies by comparing real-time measurements with predicted behaviors [10,11,12]. These techniques offer low-latency response and low computational complexity, making them suitable for embedded or real-time systems. However, their performance depends heavily on the accuracy of the system model, rendering them sensitive to modeling errors and external disturbances. Conversely, learning-based methods utilize artificial intelligence and machine learning techniques, such as neural networks (NNs) and deep learning algorithms, to identify anomalies through data-driven pattern recognition [13,14,15]. While effective in capturing complex system behaviors, these approaches require large-scale training data and impose significant computational overhead, which can limit their feasibility in safety-critical systems with constrained resources. To exploit the advantages of both approaches, hybrid strategies that integrate model-based estimation with learning-based techniques have been proposed [16], achieving improved robustness against uncertainties and enhanced anomaly detection performance [17]. Beyond anomaly detection, another class of secure control strategies involves state constraints imposed by control barrier functions (CBFs); however, like most detection-based methods, these strategies are usually time-triggered [18,19].

While secure control is essential for system reliability, it must also operate efficiently under resource constraints. Traditional time-triggered control, which updates inputs periodically regardless of system behavior, often leads to redundant computation and excessive communication. To address these inefficiencies, event-triggered control (ETC) updates control actions only when a predefined condition is violated [20,21]. This selective updating reduces unnecessary transmissions, network congestion, and processor load. While conventional ETC designs neglect resilience against cyber-attacks [22,23], a growing body of research has begun to address this gap by integrating ETC mechanisms into secure control strategies [24,25,26,27,28,29,30,31]. These efforts represent important steps toward enhancing the robustness of ETC systems in adversarial environments.

The frameworks in [24,25] propose event-driven secure control triggered only upon cyber-attack detection. While practically valuable, they lack formal stability guarantees and may cause undesirable transients or degraded performance during nominal operation, especially in systems sensitive to disturbances or uncertainties.

Other ETC-based secure control strategies have focused on mitigating the impact of DoS attacks. For instance, refs. [26,27] examined ETC mechanisms under actuator-side and joint sensor–actuator DoS attacks, respectively; however, neither approach incorporates an anomaly detection mechanism. A switching-like ETC scheme is introduced in [28], where the triggering threshold adapts based on the presence or absence of acknowledgment signals as indicators of communication status. While DoS attacks typically manifest as communication losses, FDI attacks compromise data integrity without disrupting the communication flow. As a result, detection strategies relying solely on communication feedback may be ineffective, particularly against stealthy integrity attacks.

Few studies have investigated secure ETC frameworks in the presence of FDI threats. The method in [29] proposes an ETC scheme under FDI attacks modeled by Bernoulli processes, using a state-dependent triggering mechanism and recursive linear matrix inequality (LMI) conditions; however, it lacks an observer or detection mechanism, which limits its ability to distinguish intentional manipulation from model mismatch. The authors who previously investigated DoS attacks in [28] later proposed a secure observer-based ETC method for actuator-side FDI attacks in [30]. An adaptive neural network estimates the injected signal, assumed to occur only at event-triggering instants. The triggering parameters are manually selected, and weight updates are performed exclusively at those instants. While this piecewise-constant strategy improves robustness against transmission corruption, it may slow convergence and reduce estimation accuracy when events are infrequent. The scheme in [31] addresses sensor-side FDI attacks by comparing $\chi^{2}$-based detection index against a fixed threshold. Although a Lyapunov-based stability proof is included, the triggering and detection thresholds are not formulated as decision variables.

To the best of our knowledge, a unified ETC framework that jointly enables real-time hybrid detection of sensor-side FDI attacks, systematic triggering threshold synthesis, and closed-loop stability guarantees under resource constraints remains largely underexplored in the current literature. Unlike conventional approaches, the proposed method introduces a unified framework that integrates a Kalman filter–neural network hybrid observer with a state-dependent event-triggered control strategy. The neural network component is governed by Lyapunov-based weight update laws, and the triggering threshold is derived within a linear matrix inequality framework to ensure both stability and communication-aware control. The proposed framework is validated through a range of FDI attack scenarios exhibiting diverse temporal characteristics, specifically designed to demonstrate its robustness across a wide spectrum of adversarial conditions.

The main contributions of this study are summarized as follows:

(i) Real-time hybrid anomaly detection: A hybrid detection method is developed by combining a Kalman filter with a Lyapunov-based adaptive neural network. The update laws for the neural network weights are analytically derived to ensure that the time derivative of the unified Lyapunov function remains negative definite, thereby guaranteeing the stability of both the closed-loop system and the neural network dynamics.
(ii) Resource-aware secure control: A composite control structure is designed by integrating the hybrid observer with a state-dependent event-triggered mechanism. The triggering condition is dynamically adjusted based on the estimation feedback, allowing control updates to occur only when necessary. This strategy enhances resilience while significantly reducing communication and computational overhead.
(iii) Stability-guaranteed LMI-based threshold synthesis: A unified Lyapunov function is constructed to capture both estimation and triggering dynamics. Based on this formulation, LMI conditions are derived to ensure boundedness of estimation and system errors. The triggering threshold is treated as a decision variable within the LMI framework, enabling systematic optimization for both stability assurance and resource efficiency.

The remainder of the paper is organized as follows: Section 2 formulates the system dynamics and models the FDI attacks. Section 3 introduces the ETC scheme and details the controller design. Section 4 describes the attack detection framework and the design of a hybrid observer integrating a Kalman filter and a neural network. Section 5 provides a Lyapunov-based stability analysis to ensure the boundedness of estimation and system errors, as well as to determine the triggering threshold. Section 6 demonstrates the effectiveness of the proposed method through simulation results. Finally, Section 7 concludes the paper.

## 2. Mathematical Model

A reliable detection mechanism against cyber-attacks requires a detailed characterization of the system’s behavior. Due to the nonlinear nature of robot manipulator dynamics, direct control design is often analytically intractable. To overcome this, the nonlinear equations are linearized around an operating point, yielding a simplified model that is amenable to classical control techniques. This linear approximation not only reduces computational burden but also enables structured analysis and design procedures. It forms the basis of the proposed secure control scheme, which aims to maintain stability and performance even when sensor data integrity is compromised.

### 2.1. FDI Attacks Model

False data injection attacks involve manipulating sensor signals to insert misleading information into control systems. Such deception causes the system to misinterpret abnormal situations as normal, potentially leading to inappropriate control actions. Consequently, this may eventually degrade system stability and reduce overall performance [9].

The effect of the attack can be modeled as

(1) φ(ν,α)=ν(t)+f(t)

where $\varphi \in R^{n}$ is a known linear function, $\nu \left(t\right)\in R^{n}$ is the signal under FDI attack, and $f\left(t\right)\in R^{n}$ is defined as

(2) f(t)=α(t)+θ(t)

where $\alpha \left(t\right)\in R^{n}$ denotes unknown, continuous, and bounded FDI attacks, and θ(t) represents the existing noise in the measurements. For simplicity, θ(t) is modeled as part of the FDI attacks [32].

To avoid detection, a well-designed FDI attack would typically avoid using very large or rapidly changing signals, as such behavior could be easily identified by anomaly detection mechanisms [33].

**Assumption** **A1.** *It is assumed that f(t) is bounded such that $∥f\left(t\right)∥\leq \bar{f}$ holds for all t, where $\bar{f}$ is a known positive constant.*

### 2.2. System Model Under FDI Attack

**Figure 1:**
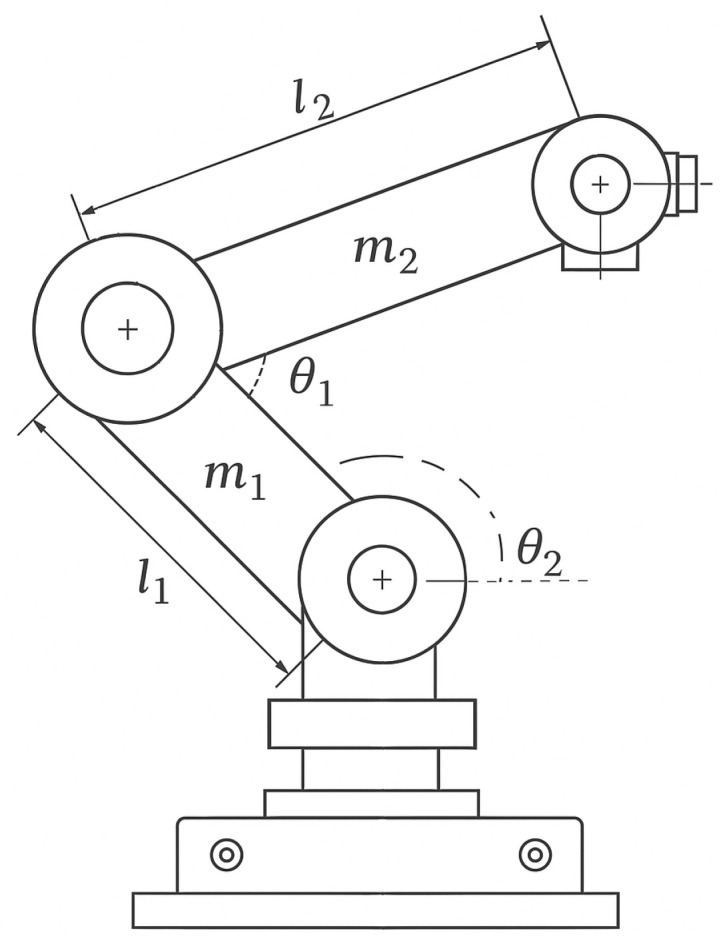
The 2-DOF robot manipulator.

The two-degree-of-freedom (2-DOF) robot manipulator system comprises two rotary joints, forming the fundamental structure for precise and flexible motion control. The arm links of the manipulator are characterized by their lengths, denoted as $l_{1}$ and $l_{2}$, and their respective masses, $m_{1}$ and $m_{2}$. The rotational motion of the joints is described by the angles $\theta_{1}$ and $\theta_{2}$, which define the system’s configuration. Figure 1 illustrates the physical structure of the 2-DOF robot manipulator, including its rotary joints, arm links, and their associated parameters.

The dynamics of a general n-degree-of-freedom robot manipulator are mathematically formulated using the Euler–Lagrange equation [34]

(3) $$M\left(q\right)\ddot{q}+V_{m}\left(q,\dot{q}\right)+G\left(q\right)+F\left(\dot{q}\right)+d\left(t\right)=T\left(t\right).$$

In this equation, $q\left(t\right)\in R^{n}$ represents the joint variable vector, $M\left(q\right)\in R^{n\times n}$ denotes the inertia matrix, $V_{m}\left(q,\dot{q}\right)\in R^{n\times n}$ is the Coriolis/centrifugal matrix, $G\left(q\right)\in R^{n}$ represents the gravity vector, $F\left(\dot{q}\right)\in R^{n}$ includes the friction terms, and $d\left(t\right)\in R^{n}$ accounts for disturbances. Additionally, $T\left(t\right)\in R^{n}$ is the system input torque.

To facilitate the use of well-established linear control methods, which are generally more computationally efficient and easier to analyze and implement than their nonlinear counterparts, the nonlinear Euler–Lagrange model is linearized around a fixed equilibrium point. The linearization is carried out using a first-order Taylor expansion, assuming sufficiently small deviations and neglecting higher-order terms. The resulting state-space model is presented in (4), with constants $C_{1}-C_{6}$ and system states defined accordingly $x_{1}\left(t\right)=\theta_{1}\left(t\right)$, $x_{2}\left(t\right)=\theta_{2}\left(t\right)$, $x_{3}\left(t\right)={\dot{\theta }}_{1}\left(t\right)$, $x_{4}\left(t\right)={\dot{\theta }}_{2}\left(t\right)$.

(4) $$\begin{array}{cc} \left[\begin{array}{c} {\dot{x}}_{1}\left(t\right)\\ {\dot{x}}_{2}\left(t\right)\\ {\dot{x}}_{3}\left(t\right)\\ {\dot{x}}_{4}\left(t\right) \end{array}\right]\; & =\left[\begin{array}{cccc} 0 & 0 & 1 & 0\\ 0 & 0 & 0 & 1\\ \frac{C_{4}C_{5}}{C_{1}C_{5}} & \frac{C_{4}C_{6}}{C_{1}C_{5}} & 0 & 0\\ 0 & \frac{-C_{6}}{C_{5}} & 0 & 0 \end{array}\right]\left[\begin{array}{c} x_{1}\left(t\right)\\ x_{2}\left(t\right)\\ x_{3}\left(t\right)\\ x_{4}\left(t\right) \end{array}\right]+\left[\begin{array}{cc} 0 & 0\\ 0 & 0\\ \frac{C_{5}}{C_{1}C_{5}} & \frac{-C_{2}}{C_{1}C_{5}}\\ 0 & \frac{1\;-\;C_{2}}{C_{5}} \end{array}\right]\left[\begin{array}{c} T_{1}\left(t\right)\\ T_{2}\left(t\right) \end{array}\right]\\ y(t) & =Cx(t)+f(t) \end{array}$$

Defining an FDI attack at the sensor introduces an additional term, f(t), in the measurement equation, representing the influence of the attack on the system. Such an attack has the potential to destabilize the system or significantly degrade its performance [35]. The FDI attack is mathematically modeled as a function $\phi :\left[t_{0},\infty \right)\to R^{n}$, which alters the measured feedback signals in the following manner

(5) $$\begin{array}{cc} y(t) & =\varphi \left(x\left(t\right),f_{1}\left(t\right)\right)=x\left(t\right)+f_{1}\left(t\right)\\ \dot{y}\left(t\right) & =\varphi \left(\dot{x}\left(t\right),f_{2}\left(t\right)\right)=\dot{x}\left(t\right)+f_{2}\left(t\right) \end{array}$$

where $f_{j}\left(t\right)=\alpha_{j}\in R^{n}\;j=\left\{1,2\right\}$ represents FDI attacks that are unknown, bounded, time-varying, and continuous, directly impacting the system states.

## 3. Event-Triggered Control

To reduce unnecessary control updates while maintaining closed-loop stability in the presence of FDI attacks targeting sensor measurements, we propose an ETC strategy. In this framework, the control input remains constant between trigger instants and is updated only when a predefined event condition, based on the estimated state of the system, is violated. This design ensures resource efficiency while preserving performance under adversarial conditions.

Let $t_{k}\in \left\{t_{0},t_{1},\ldots \right\}$ be the time instants when an event is triggered [36]. The observer estimation error is then introduced to formulate the event-triggering criterion, defined as [37]

(6) $$e_{\hat{x}}\left(t\right)=\hat{x}\left(t_{k}\right)-\hat{x}\left(t\right),\;t\in \left[t_{k},t_{k+1}\right),\;k\in Z_{0}^{+}.$$

The observer estimation error $e_{\hat{x}}\left(t\right)$ represents the difference between the last transmitted estimate and the current observer output.

An event is triggered whenever the squared norm of the observer error surpasses the threshold defined in

(7) $$∥e_{\hat{x}}{\left(t\right)∥}^{2}<\sigma {∥\hat{x}\left(t\right)∥}^{2},\;t\in \left[t_{k},t_{k+1}\right)$$

which implies that the next triggering instant $t_{k+1}$ is determined once the inequality in (7) is violated [38]. The scalar σ>0 is the design parameter that governs the trade-off between control update frequency and system performance. Rather than selecting it heuristically, this work computes σ by solving a linear matrix inequality derived from Lyapunov-based stability conditions, ensuring formal guarantees.

**Remark** **1.** *The proposed event-triggering condition is designed to prevent Zeno behavior by ensuring that the time between two triggering instants is always positive. At each triggering time $t_{k}$, the triggering error $e_{\hat{x}}\left(t\right)$ is reset to zero. If $\hat{x}\left(t\right)\neq 0$, the triggering condition is not satisfied immediately, and a positive time interval must pass before the next triggering occurs. Since $\hat{x}\left(t\right)$ is continuous, the triggering condition cannot be satisfied infinitely often in a finite time. If $\hat{x}\left(t\right)=0$, the triggering condition may be violated, but this indicates that the system is in a steady state and no further control updates are needed.*

The control input u(t) is designed to be updated only at the event-triggered instants ${\left\{t_{k}\right\}}_{k=0}^{\infty }$ according to

(8) $$u\left(t\right)=K\hat{x}\left(t_{k}\right),\;t\in \left[t_{k},t_{k+1}\right)$$

where *K* is the state feedback gain matrix, designed using the Linear Quadratic Regulator (LQR) method to ensure closed-loop stability and desired performance [39,40]. The signal $x(t_{k})$ represents the estimated state at the last triggering instant. The control input is held constant between triggering times using a zero-order-hold (ZOH) mechanism [36].

## 4. Attack Detection

In this section, a neural network-based observer framework is proposed for attack detection. A Kalman filter is first employed to estimate the system states. Given the absence of an explicit attack model and the inherently complex and uncertain nature of cyber–physical attacks, neural networks serve as powerful function approximators [41]. Thus, they are well-suited to estimate such anomalies.

### 4.1. Observer Design

An observer is designed to provide accurate estimations of system states, which are essential for implementing effective control strategies. Together, the linearized model, secure control framework, and state observer form a cohesive approach to addressing sensor attacks in robot manipulators, which are integral to autonomous intelligent systems operating in safety-critical environments.

Given that cyber-attacks can severely degrade control performance, reliable state estimation becomes imperative. To address this issue, an estimator based on the Kalman filter is employed to provide accurate state information under model and measurement uncertainties. The Kalman filter is a robust and widely adopted algorithm in control applications [42]. Its primary advantage lies in its capacity to fuse model-based predictions with real-time measurement data, effectively mitigating the impact of noise and uncertainty. In systems where both the process dynamics and measurement models exhibit linear behavior, the Kalman filter provides optimal state estimation in the minimum mean-square error sense [43]. In this study, it is used to refine the state prediction, ensuring the accuracy needed for both attack detection and controller execution. The mathematical representation of the estimated state dynamics is

(9) $$\begin{array}{cc} \dot{\hat{x}}\left(t\right) & =A\hat{x}\left(t\right)+Bu\left(t\right)+L\left(y\left(t\right)-\hat{y}\left(t\right)\right)\\ \hat{y}\left(t\right) & =C\hat{x}\left(t\right)+\hat{f}\left(t\right). \end{array}$$

**Figure 2:**
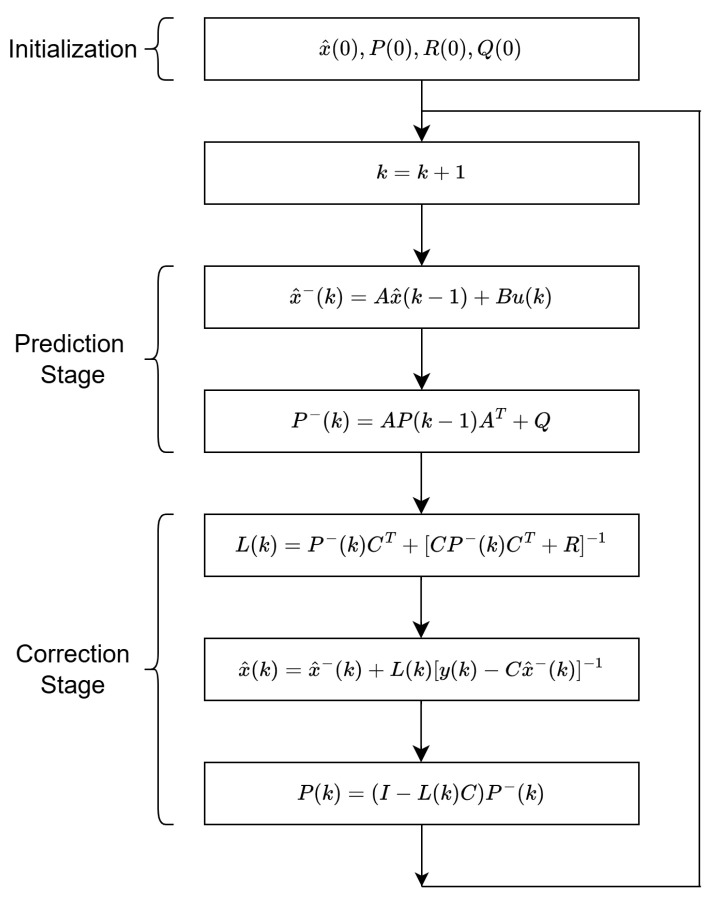
Algorithm of Kalman filter.

The determination of the Kalman filter gain plays a vital role in achieving high-accuracy state estimation. The operational principle of the Kalman filter is depicted in Figure 2. According to this principle, L(k) represents the Kalman filter gain, $P^{-}\left(k\right)$ denotes the prior covariance matrix of the state prediction error, and P(k) represents the updated covariance matrix of the state prediction error. Q(k) and R(k) are the covariance matrices of the process and measurement noise, respectively. Here, *k* indicates the sampling instance, and $T_{s}$ represents the sampling time, with the relationship given as $k=t/T_{s}$. At each sampling instance, the system states are iteratively updated following the algorithm presented in Figure 2, ensuring more accurate and reliable state estimation.

The output estimation error is defined as

(10) $$e\left(t\right):=y\left(t\right)-\hat{y}\left(t\right)$$

which serves as a critical input for the neural network-based observer, enabling the detection and mitigation of FDI.

### 4.2. Neural Network

To estimate the unknown attack signal affecting sensor measurements, a three-layer feedforward neural network is introduced. The neural network functions as a universal approximator to estimate the adversarial signal in real time [44]. However, the universal approximation theorem is guaranteed to hold only over a compact domain [45]. Since FDI attacks may occur over a non-compact time domain, a nonlinear mapping is required to project time into a compact spatial domain as proposed in [46]. Let $M_{f}:t\to \Theta$ be defined as

(11) $$M_{f}≜\frac{\kappa_{f}t}{1+\kappa_{f}t},\;t\in \left[0,\infty \right),\;\Theta \in \left[0,1\right],$$

where $\kappa_{f}\in R^{+}$ is a user-defined saturation coefficient. The function f(t) can be mapped into the compact domain Θ as

(12) $$f\left(t\right)=f\left(M_{f}^{-1}\left(\Theta \right)\right)=f_{M_{f}}\left(\Theta \right).$$

According to the universal function approximation theorem, $f_{M_{f}}\left(\Theta \right)$ can be represented as

(13) $$f_{M_{f}}\left(\Theta \right)=W^{⊤}\sigma \left(V^{⊤}\delta \right)+\varepsilon .$$

Let $N_{1}$, $N_{2}$, and $N_{3}$ denote the number of neurons in the input, hidden, and output layers, respectively. The NN weights are given by $W\in R^{\left(N_{2}+1\right)\times N_{3}}$ and $V\in R^{\left(N_{1}+1\right)\times \left(N_{2}\right)}$. The activation function is denoted by $\sigma \left(.\right)\in R^{N_{2}+1}$. The reconstruction error is $\epsilon \in R^{N_{3}}$. The input vector $\delta \left(t\right)\in R^{\left(N_{1}+1\right)}$ is constructed using previous estimates of the FDI attack and output error signals.

(14) $$\delta \left(t\right)={\left[1,{\hat{f}}^{⊤}\left(t-T_{s}\right),\ldots ,{\hat{f}}^{⊤}\left(t-aT_{s}\right),e^{⊤}\left(t-T_{s}\right),\ldots ,e^{⊤}\left(t-bT_{s}\right)\right]}^{⊤}.$$

Note that augmenting the input vector δ and activation function σ by “1” allows us to have thresholds as the first columns of the weight matrices. Thus, any tuning of W and V then includes tuning of thresholds as well [47].

**Figure 3:**
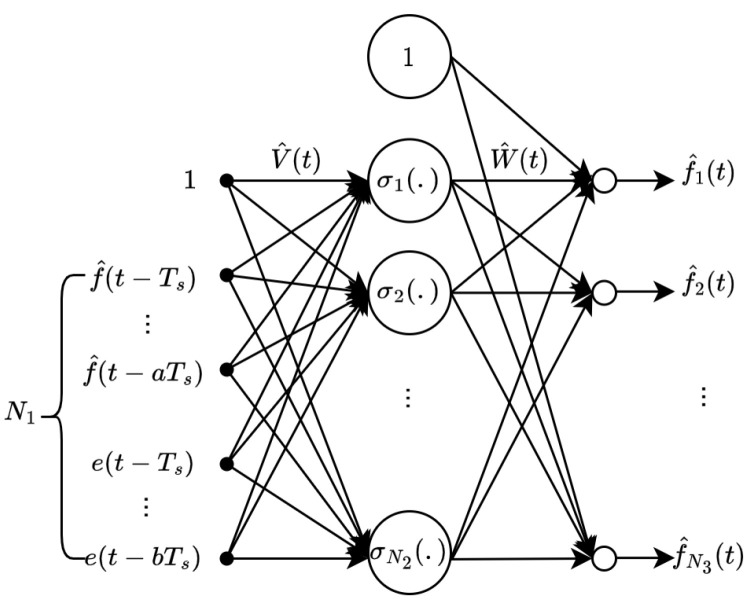
The structure of the neural network.

Based on (13), the output of the NN estimator is

(15) $$\hat{f}\left(t\right)={\hat{W}}^{⊤}\sigma \left({\hat{V}}^{⊤}\delta \right)$$

where $\hat{W}$ and $\hat{V}$ are the current estimates of the ideal weights *W* and *V*. To complement the mathematical formulation, the structure of the NN is illustrated in Figure 3.

Depicted in Figure 3, the network processes the input vector δ(t). The structure consists of two weight matrices, $\hat{W}$ and $\hat{V}$, which are updated online using projection-based adaptation laws. The output nodes represent the individual components of the attack estimate vector $\hat{f}\left(t\right)\in R^{N_{3}}$. The NN output serves as an estimate of the unknown adversarial signal.

The mismatch between f(t) and $\hat{f}\left(t\right)$ can be approximated using a Taylor’s series approximation, which, after some algebraic manipulation, can be expressed as

(16) $$\tilde{f}={\tilde{W}}^{⊤}\sigma \left({\hat{V}}^{⊤}\delta \right)+{\hat{W}}^{⊤}\sigma^{\prime }\left({\hat{V}}^{⊤}\delta \right){\tilde{V}}^{⊤}\delta +N$$

where the residual term is

(17) $$N≜{\tilde{W}}^{⊤}\sigma^{\prime }\left({\hat{V}}^{⊤}\delta \right){\tilde{V}}^{⊤}\delta +W^{⊤}O\left({\tilde{V}}^{⊤}\delta_{i}\right)+\varepsilon .$$

Here, $\sigma^{\prime }\left(V^{⊤}\delta \right)=\partial \sigma \left(V^{⊤}\delta \right)/\partial \left(V^{⊤}\delta \right)$ evaluated at ${\hat{V}}^{⊤}\delta$, $\tilde{W}=W-\hat{W}$ and $\tilde{V}=V-\hat{V}$ denote the outer and inner NN weight error, respectively. O denotes higher-order terms and $∥N∥\leq \bar{n}$ for some $\bar{n}\in R_{>0}$ [48].

To minimize the estimation error defined in (16), and in accordance with the subsequent Lyapunov-based stability analysis, the update laws for the estimated weights $\hat{W}$ and $\hat{V}$ are designed as

(18) $$\begin{array}{cc} \dot{\hat{W}} & =proj\left(\Gamma_{W}\;\sigma \left({\hat{V}}^{⊤}\delta \right)\left(L^{⊤}P_{2}\hat{x}\right)\right), \end{array}$$

(19) $$\begin{array}{cc} \dot{\hat{V}} & =proj\left(\Gamma_{V}\;\delta {\left(L^{⊤}P_{2}\hat{x}\right)}^{⊤}{\hat{W}}^{⊤}\sigma^{\prime }\left({\hat{V}}^{⊤}\delta \right)\right) \end{array}$$

where $\Gamma_{W}$ and $\Gamma_{V}$ are positive definite learning rates. To ensure that $\hat{W}$ and $\hat{V}$ remain bounded during adaptation, the projection operator proj(.) is applied as defined in [49].

**Figure 4:**
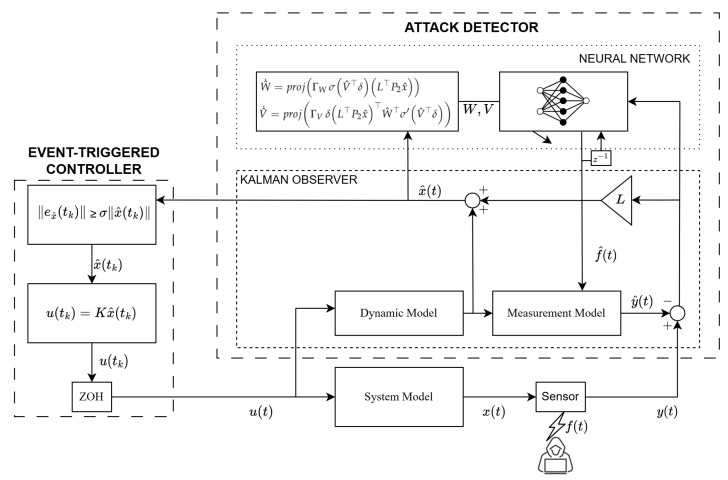
The architecture of the whole system under FDI attack.

The architecture of the whole system under attack is shown in Figure 4, which summarizes the structure of the proposed event-triggered secure control system, including the hybrid observer, controller, and attack channel. The subsequent section provides the corresponding stability analysis.

## 5. Stability Analysis

In this section, the stability analysis of the proposed event-triggered secure control method is presented. To ensure that the system operates robustly and reliably in the presence of sensor attacks, Lyapunov stability analysis is utilized to evaluate the system’s stability.

The following lemmas are used in the stability analysis of the closed-loop system.

**Lemma** **1.** *Let x and y be matrices of compatible sizes. Then, the following inequality holds*

(20) $$X^{⊤}Y+Y^{⊤}X\leq X^{⊤}X+Y^{⊤}Y.$$

**Lemma** **2.** *The attack signal estimation error is upper-bounded by the following inequality*

(21) $$∥\tilde{f}\left(t\right)∥\leq U_{f}∥\tilde{x}\left(t\right)∥$$

*where $U_{f}$ denotes a positive constant [50].*

Let *S* be defined as

(22) $$S≜\frac{1}{2}\mathrm{tr}\left({\tilde{W}}^{⊤}\Gamma_{W}^{-1}\tilde{W}\right)+\frac{1}{2}\mathrm{tr}\left({\tilde{V}}^{⊤}\Gamma_{V}^{-1}\tilde{V}\right)$$

where $S:\left[t_{0},\infty \right)\to R_{\geq 0}$.

**Remark** **2.** *Since $\tilde{W}$ and $\tilde{V}$ are bounded; therefore, S is bounded, i.e., $\left|S\right|\leq S_{\max}$, where $S_{\max}\;\in \;R_{>0}$ is a known positive constant.*

**Theorem** **1.** *If there exist the matrices $P_{1}>0$, $P_{2}>0$, $P_{3}>0$ and constants γ>0, σ>0 which satisfy the following LMIs*

(23) $$\left[\begin{array}{ccccc} \Phi_{1} & 0 & -\frac{1}{2}P_{1}BK & P_{1}BK & 0\\ {}* & \Phi_{2} & \frac{1}{2}P_{2}LC & 0 & P_{2}BK\\ {}* & * & \Phi_{3} & 0 & 0\\ {}* & * & * & -I & 0\\ {}* & * & * & * & -I \end{array}\right]<0$$

*where*

(24) $$\begin{array}{cc} \Phi_{1} & =\frac{1}{2}\left[{\left(A+BK\right)}^{⊤}P_{1}+P_{1}\left(A+BK\right)\right], \end{array}$$

(25) $$\begin{array}{cc} \Phi_{2} & =\frac{1}{2}\left[{\left(A+BK\right)}^{⊤}P_{2}+P_{2}\left(A+BK\right)+2\sigma I\right], \end{array}$$

(26) $$\begin{array}{cc} \Phi_{3} & =\frac{1}{2}\left[2U_{f}\gamma +{\left(A-LC\right)}^{⊤}P_{3}+P_{3}\left(A-LC\right)\right], \end{array}$$

*and subject to norm bound*

(27) $$\left[\begin{array}{cc} \gamma I & P_{3}L\\ L^{⊤}P_{3} & \gamma I \end{array}\right]>0$$

*then the system (4), controlled via the event-triggered controller (8), which is based on observer (9) and neural network (15), is guaranteed to be uniformly ultimately bounded (UUB).*

**Proof.**  A Lyapunov-based approach is used for the stability analysis of the closed-loop system. Firstly, we define observer error, which is

(28) $$\tilde{x}\left(t\right)=x\left(t\right)-\hat{x}\left(t\right).$$

Then, the closed-loop system dynamics are constructed according to event-trigger-based control,

(29) $$\begin{array}{cc} \dot{x}\left(t\right) & =Ax\left(t\right)+BK\hat{x}\left(t_{k}\right)=\left(A+BK\right)x\left(t\right)-BK\tilde{x}\left(t\right)+BKe_{\hat{x}}\left(t\right), \end{array}$$

(30) $$\begin{array}{cc} \dot{\hat{x}}\left(t\right) & =\left(A+BK\right)\hat{x}\left(t\right)+BKe_{\hat{x}}\left(t\right)+LC\tilde{x}\left(t\right)+L\left(\tilde{f}\left(t\right)\right), \end{array}$$

(31) $$\begin{array}{cc} \dot{\tilde{x}}\left(t\right) & =\left(A-LC\right)\tilde{x}\left(t\right)-L\tilde{f}\left(t\right). \end{array}$$

The candidate Lyapunov function is proposed as

(32) $$V_{L}=\frac{1}{2}\left[x^{⊤}\left(t\right)P_{1}x\left(t\right)+{\hat{x}}^{⊤}\left(t\right)P_{2}\hat{x}\left(t\right)+{\tilde{x}}^{⊤}\left(t\right)P_{3}\tilde{x}\left(t\right)\right]+S.$$

Subsequently, the derivative of the candidate Lyapunov function is derived as

(33) $$\begin{array}{cc} \dot{V_{L}}\left(t\right) & =x^{⊤}P_{1}\dot{x}+{\hat{x}}^{⊤}P_{2}\dot{\hat{x}}+{\tilde{x}}^{⊤}P_{3}\dot{\tilde{x}}+tr\left({\tilde{W}}^{⊤}\Gamma_{W}^{-1}\dot{\tilde{W}}\right)+tr\left({\tilde{V}}^{⊤}\Gamma_{V}^{-1}\dot{\tilde{V}}\right). \end{array}$$

Considering the fact that $\dot{\tilde{W}}=-\dot{\hat{W}}$ and $\dot{\tilde{V}}=-\dot{\hat{V}}$, and substituting (29)–(31) into (33)

(34) $$\begin{array}{cc} \dot{V_{L}}\left(t\right) & =x^{⊤}P_{1}\left(A+BK\right)x-x^{⊤}P_{1}BK\tilde{x}+x^{⊤}P_{1}BKe_{\hat{x}}\\ \; & \;+{\hat{x}}^{⊤}P_{2}\left(A+BK\right)\hat{x}+{\hat{x}}^{⊤}P_{2}BKe_{\hat{x}}+{\hat{x}}^{⊤}P_{2}LC\tilde{x}+{\hat{x}}^{⊤}P_{2}L\tilde{f}\\ \; & \;+{\tilde{x}}^{⊤}P_{3}\left(A-LC\right)\tilde{x}-{\tilde{x}}^{⊤}P_{3}L\tilde{f}-tr\left({\tilde{W}}^{⊤}\Gamma_{W}^{-1}\dot{\hat{W}}\right)-tr\left({\tilde{V}}^{⊤}\Gamma_{V}^{-1}\dot{\hat{V}}\right). \end{array}$$

Substituting (16) into (34) and then applying (18) and (19) cancels the cross-terms. Furthermore, according to Lemma 1, an upper bound on the derivative of the candidate Lyapunov function can be defined as

(35) $$\begin{array}{cc} \dot{V_{L}} & \leq \frac{1}{2}x^{⊤}\left({\left(A+BK\right)}^{⊤}P_{1}+P_{1}\left(A+BK\right)+P_{1}BKK^{⊤}B^{⊤}P_{1}\right)x-x^{⊤}P_{1}BK\tilde{x}\\ \; & \;+\frac{1}{2}{\hat{x}}^{⊤}\left({\left(A+BK\right)}^{⊤}P_{2}+P_{2}\left(A+BK\right)+P_{2}BKK^{⊤}B^{⊤}P_{2}\right)\hat{x}+e_{\hat{x}}^{⊤}e_{\hat{x}}\\ \; & \;+{\hat{x}}^{⊤}P_{2}LC\tilde{x}+\frac{1}{2}{\tilde{x}}^{⊤}\left({\left(A-LC\right)}^{⊤}P_{3}+P_{3}\left(A-LC\right)\right)\tilde{x}-{\tilde{x}}^{⊤}P_{3}L\tilde{f}+{\hat{x}}^{⊤}P_{2}L\bar{n}. \end{array}$$

To transform the stability condition into a standard LMI framework, it is necessary to derive appropriate upper bounds for the terms $e_{\hat{x}}^{⊤}e_{\hat{x}}$, $-{\tilde{x}}^{⊤}P_{3}L\tilde{f}$ and ${\hat{x}}^{⊤}P_{2}L\bar{n}$ in (35). According to event condition (7), $∥e_{\hat{x}}{\left(t\right)∥}^{2}<\sigma {∥\hat{x}\left(t\right)∥}^{2}$ holds for all t≥0. Using Lemma 2, the upper bound for $-{\tilde{x}}^{⊤}P_{3}L\tilde{f}$ is given as follows:

(36) $$-{\tilde{x}}^{⊤}P_{3}L\tilde{f}\leq |{\tilde{x}}^{⊤}P_{3}L\tilde{f}|\leq ∥{\tilde{x}}^{⊤}∥∥P_{3}L∥∥\tilde{f}∥\leq U_{f}∥P_{3}L∥|\tilde{x}{∥}^{2}.$$

The upper bound for the last term is defined as

(37) $${\hat{x}}^{⊤}P_{2}L\bar{n}\leq \Psi$$

where Ψ depends on the prediction error of system states for the observer of the and neural network attack estimation error. Substituting (36) and (37) into (35)

(38) $$\begin{array}{cc} \dot{V_{L}} & \leq \frac{1}{2}x^{⊤}\left({\left(A+BK\right)}^{⊤}P_{1}+P_{1}\left(A+BK\right)+P_{1}BKK^{⊤}B^{⊤}P_{1}\right)x-x^{⊤}P_{1}BK\tilde{x}\\ \; & \;+\frac{1}{2}{\hat{x}}^{⊤}\left({\left(A+BK\right)}^{⊤}P_{2}+P_{2}\left(A+BK\right)+P_{2}BKK^{⊤}B^{⊤}P_{2}+2\sigma I\right)\hat{x}\\ \; & \;+{\hat{x}}^{⊤}P_{2}LC\tilde{x}+\frac{1}{2}{\tilde{x}}^{⊤}\left({\left(A-LC\right)}^{⊤}P_{3}+P_{3}\left(A-LC\right)+∥P_{3}L∥U_{f}\right)\tilde{x}+\Psi . \end{array}$$

To manage the nonlinearity introduced by $∥P_{3}L∥$, we impose the following bound:

(39) $$∥P_{3}L∥<\gamma .$$

Accordingly, the derivative of the Lyapunov function is upper bounded as follows:

(40) $$\begin{array}{cc} \dot{V_{L}} & \leq \frac{1}{2}x^{⊤}\left({\left(A+BK\right)}^{⊤}P_{1}+P_{1}\left(A+BK\right)+P_{1}BKK^{⊤}B^{⊤}P_{1}\right)x-x^{⊤}P_{1}BK\tilde{x}\\ \; & \;+\frac{1}{2}{\hat{x}}^{⊤}\left({\left(A+BK\right)}^{⊤}P_{2}+P_{2}\left(A+BK\right)+P_{2}BKK^{⊤}B^{⊤}P_{2}+2\sigma I\right)\hat{x}\\ \; & \;+{\hat{x}}^{⊤}P_{2}LC\tilde{x}+\frac{1}{2}{\tilde{x}}^{⊤}\left({\left(A-LC\right)}^{⊤}P_{3}+P_{3}\left(A-LC\right)+\gamma U_{f}\right)\tilde{x}+\Psi . \end{array}$$

Finally, by defining the augmented state vector as

(41) $$z={\left[\begin{array}{c} x^{⊤},{\hat{x}}^{⊤},{\tilde{x}}^{⊤} \end{array}\right]}^{⊤},$$

the derivative of the Lyapunov function is given by

(42) $$\dot{V_{L}}\leq z^{⊤}\Phi z+\Psi .$$

The augmented matrix Φ is given by

(43) $$\Phi =\left[\begin{array}{ccc} \Phi_{1}+P_{1}BKK^{⊤}B^{⊤}P_{1} & 0 & -\frac{1}{2}P_{1}BK\\ {}* & \Phi_{2}+P_{2}BKK^{⊤}B^{⊤}P_{2} & \frac{1}{2}P_{2}LC\\ {}* & * & \Phi_{3} \end{array}\right]$$

where Φ is a symmetric matrix constructed from the cross terms and quadratic terms derived earlier. By applying the Schur complement to (43), one obtains the equivalent LMI condition given in (23). Moreover, the norm bound on $∥P_{3}L∥$ is also incorporated as an additional LMI constraint. □

## 6. Simulation Results

Simulation studies are conducted on a 2-DOF robot manipulator with a sampling time of $10^{-3}$ s. The state-space model matrices, derived from the system model defined in (4) and utilizing the parameter values provided in the literature [51], are presented as

(44) $$\begin{array}{cc} A & =\left[\begin{array}{cccc} 0 & 0 & 1 & 0\\ 0 & 0 & 0 & 1\\ -0.4568 & -0.6196 & 0 & 0\\ 0.2485 & -6.6174 & 0 & 0 \end{array}\right],\;B=\left[\begin{array}{cc} 0 & 0\\ 0 & 0\\ 0.7870 & -0.0426\\ 0.04285 & 0.1349 \end{array}\right],\;C=\left[\begin{array}{cccc} 1 & 0 & 0 & 0\\ 0 & 1 & 0 & 0\\ 0 & 0 & 1 & 0\\ 0 & 0 & 0 & 1 \end{array}\right]. \end{array}$$

The Kalman observer gain *L* and the LQR gain *K* are

(45) $$L=\left[\begin{array}{cccc} 44.7264 & 0.0027 & 0.2715 & 0.1240\\ 0.0027 & 44.5708 & -0.3079 & -2.7884\\ 0.2715 & -0.3079 & 44.7210 & 0.0321\\ 0.1240 & -2.7884 & 0.0321 & 45.0464 \end{array}\right],$$

(46) $$K=\left[\begin{array}{cccc} 69.9087 & 3.8982 & 71.6448 & 6.7264\\ -5.1261 & 37.2290 & -6.7632 & 74.1650 \end{array}\right].$$

A three-layer feedforward neural network is used to estimate the attack. The network consists of 16 input neurons, 5 hidden neurons with a hyperbolic tangent activation function, and 4 output neurons with linear activation. There is no offline training; all weight updates are performed online via Lyapunov-based adaptive update laws. To enhance the convergence rate of the estimator, which is critical for maintaining desirable closed-loop transient performance, the design parameters of the update law are selected via a genetic algorithm.

FDI attacks can manifest in different ways depending on the attacker’s strategy and the vulnerability of the system. Based on their temporal characteristics, this study considers three representative types of attacks: abrupt, incipient, and triangle-shaped. This classification enables a comprehensive assessment of the system’s resilience to both abrupt and progressively evolving FDI attacks. The system’s performance under each scenario is discussed below.

**Case 1: Abrupt Attack.** *Abrupt attacks involve sudden, step-like deviations that cause immediate disruption in system behavior. In this scenario, a constant bias is injected into the second state from the beginning of the simulation. The injected signal is defined as follows*
  (47) $$f\left(t\right)=\left\{\begin{array}{cc} 0, & t<0\\ 0.2, & t\geq 0 \end{array}\right..$$
**Case 2: Incipient Attack.** *Incipient attacks evolve slowly over time, often staying below detection thresholds and mimicking benign signal variations. The injected bias is modeled as the step response of a first-order system*
  (48) $$f\left(t\right)=\left\{\begin{array}{cc} 0, & t<0\\ 0.2\left(1-e^{-0.5t}\right), & t\geq 0 \end{array}\right..$$
**Case 3: Triangle Attack.** *Triangle attacks feature a linearly increasing injection followed by a linear decay, bridging the behavioral characteristics between abrupt and incipient faults. The injected signal is defined as*
  (49) $$f\left(t\right)=\left\{\begin{array}{cc} 0, & t<12\\ 0.3846(t-12), & 12\leq t<25\\ 0.3846(38-t), & 25\leq t<38\\ 0, & t\geq 38 \end{array}\right..$$

**Figure 5:**
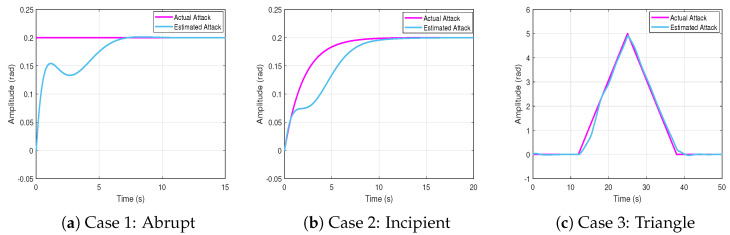
Actual and estimated attacks.

The actual and estimated attacks are shown in Figure 5 for all three scenarios.

The root mean square error (RMSE) between the actual and estimated attack is

(50) $$\mathrm{RMSE}=\sqrt{\frac{1}{N}\sum_{t=1}^{N}{\left(f\left(t\right)-\hat{f}\left(t\right)\right)}^{2}}$$

where *N* is the total number of sampling times. The proposed neural network-based estimator effectively tracks both abrupt and gradually evolving FDI profiles. In Case 1, the estimator promptly converges to the step disturbance despite its sudden onset. In Case 2, it accurately captures the slowly increasing bias with minimal tracking delay. In Case 3, the estimator successfully follows both the rising and falling trends of the attack, demonstrating its adaptability to time-varying injection patterns. These observations are also supported by the RMSE values reported in Table 1.

**Figure 6:**
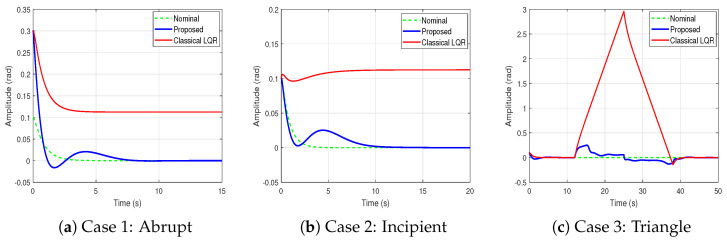
Behaviour of 2^nd^ system state under different FDI attack scenarios.

To evaluate the impact of different FDI attack profiles on closed-loop performance, three representative scenarios—abrupt, incipient, and triangle-shaped—are simulated. The corresponding system responses are illustrated in Figure 6, where each plot compares three control configurations: the nominal response (ideal case without attack), the classical LQR controller (without any protective mechanism), and the proposed scheme (which integrates a hybrid estimator and an event-triggered controller). In Case 1, the classical controller exhibits a sharp deviation due to the sudden disturbance, whereas the proposed controller promptly restores the desired trajectory. In Case 2, involving a gradually increasing bias, the proposed method maintains stable tracking performance, in contrast to the classical controller, which experiences growing deviation. In Case 3, the proposed approach effectively compensates for both the rising and falling phases of the disturbance, preserving system performance and ensuring bounded behavior. These qualitative observations are quantitatively supported by the ISE values summarized in Table 2. The results demonstrate that the proposed control framework consistently achieves improved tracking accuracy by closely following the nominal output trajectory under diverse FDI attack scenarios.

**Figure 7:**
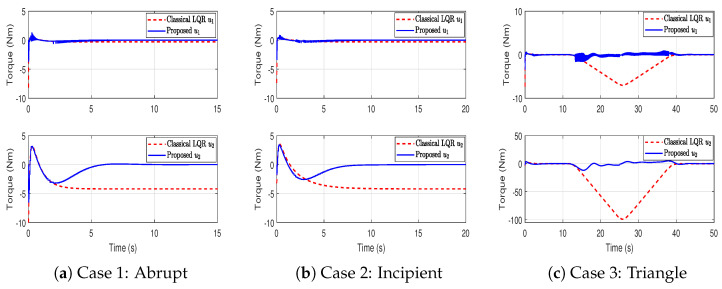
Control signals of classical LQR and proposed method under different FDI attack scenarios.

To assess the control effort associated with each method, the corresponding control signals are presented in Figure 7. Compared to the classical LQR controller, the proposed method generates smoother control actions with reduced amplitude, avoiding actuator saturation. Furthermore, Table 3 reports the control energy in terms of ISE, where the proposed scheme consistently achieves lower values, demonstrating its efficiency in minimizing control effort while preserving closed-loop performance.

**Figure 8:**
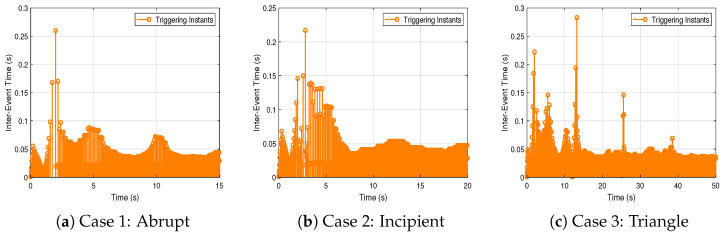
Inter-event times under different FDI attack scenarios.

The event-triggering condition is designed based on the parameter σ, computed using the MATLAB R2018b LMI Toolbox. The resulting inter-event times for each scenario are illustrated in Figure 8, where the minimum inter-event time across all cases is 0.0010 s. The average inter-event times are 0.0369, 0.0389, and 0.0353 s for the abrupt, incipient, and triangle attack scenarios, respectively. Table 4 summarizes the number of control signal transmissions. Compared to the time-triggered approach, the proposed event-triggered scheme achieves a reduction of 97.29%, 97.42%, and 97.17% in Cases 1–3, significantly decreasing communication load while maintaining control performance. This confirms that the proposed mechanism ensures strictly positive inter-event intervals and enables efficient resource utilization under various FDI attacks.

To evaluate the computational cost, the average CPU time per control step is calculated using built-in timing utilities provided by the simulation environment. The experiment is repeated several times to account for variability, and the average value is reported. As shown in Table 5, although the proposed method incurs slightly higher per-step computation time compared to the periodic approach, it demonstrates the potential of the framework for energy-efficient embedded implementations.

**Remark** **3.** *To assess the real-time feasibility of the proposed method, we measure the execution time of each control cycle in the simulation environment. The implementation is performed in MATLAB/Simulink on a desktop computer equipped with an Intel Core i7 processor (2.80 GHz, 16 GB RAM). Under a 1 ms sampling interval, the combined computation time for the observer update, neural network weight adaptation, and control signal calculation is approximately 0.2 ms per cycle. Although the control input is updated only at event-triggered instants and held constant otherwise, the computations are performed at each step. The neural network operates online using a lightweight projection-based adaptation law, without backpropagation or batch processing. Its structure is selected through offline optimization using a genetic algorithm and remains fixed during execution. These properties suggest that the proposed method is compatible with real-time implementation on embedded platforms equipped with sufficient processing capabilities, such as those based on high-end microcontrollers or FPGA-integrated systems.*

## 7. Conclusions

This study presents an event-triggered secure control framework to address false data injection attacks targeting sensor measurements in robotic manipulators. Unlike conventional secure control strategies that rely on continuous monitoring and frequent control updates, the proposed method integrates a state-dependent event-triggered mechanism that selectively updates the control input only when necessary, significantly reducing communication and computational overhead. A hybrid observer, combining a model-based Kalman filter with a learning-based neural network, is designed to compensate for malicious sensor anomalies in real time. In addition, a Lyapunov-based update law is designed for the neural network weights to preserve closed-loop stability. The stability of the overall closed-loop system is analytically guaranteed through a Lyapunov-based analysis, supported by linear matrix inequality conditions that ensure the boundedness of estimation and system errors and provide a triggering threshold for the event-triggered mechanism.

Simulation results on a two-degree-of-freedom robot manipulator validate the effectiveness of the proposed secure control framework against three representative false data injection attack scenarios: abrupt, incipient, and triangle-shaped. In all cases, the proposed method successfully detects the injected attacks and restores the nominal system trajectory. The root mean square error between the actual and estimated attack is $1.45\times 10^{-4}$ in the abrupt case, $1.36\times 10^{-4}$ in the incipient case and $1.44\times 10^{-3}$ in the triangle-shaped case. Moreover, the control signal transmissions are reduced by 97.29%, 97.42%, and 97.17%, respectively, demonstrating the effectiveness of the event-triggered mechanism. These results confirm that the proposed framework achieves robust fault estimation and efficient control, making it suitable for resource-constrained embedded systems exposed to adversarial threats.

Beyond industrial robotics, the proposed framework can be applied to a broader range of cyber–physical systems such as autonomous vehicles, aerospace systems, and medical robotics, where secure operation and computational efficiency are essential. Many of these systems can be described by Euler–Lagrange dynamics or similar mechanical modeling approaches, which makes them suitable for applying linear control and estimation methods based on local approximations. The method developed in this study combines hybrid state estimation with neural-network-based compensation and event-triggered feedback. It relies on general principles from control theory, including observer design and Lyapunov-based stability analysis. This opens the door to adapting the same structure for a wider class of systems where real-time performance, robustness against sensor attacks, and model-based estimation are critical requirements.

## Figures and Tables

**Table 1:** RMSE between the estimated and actual attack signals.

Attack Type	RMSE
Case 1: Abrupt	$1.45\times 10^{-4}$
Case 2: Incipient	$1.36\times 10^{-4}$
Case 3: Triangle	$1.44\times 10^{-3}$

**Table 2:** ISE values of controlled outputs under different FDI scenarios.

Controller	Scenario	ISE
	Case 1: Abrupt	0.2436
Classical LQR	Case 2: Incipient	0.2398
	Case 3: Triangle	70.17
	Case 1: Abrupt	0.02497
Proposed Method	Case 2: Incipient	0.005807
	Case 3: Triangle	0.02823

**Table 3:** ISE values of control signals under different FDI attack scenarios.

Controller	Case	$u_{1}$	$u_{2}$
	Case 1: Abrupt	1.776	235.8
Classical LQR	Case 2: Incipient	1.938	284.9
	Case 3: Triangle	472.6	94,040
	Case 1: Abrupt	0.6134	25.2
Proposed Method	Case 2: Incipient	0.489	21.79
	Case 3: Triangle	6.258	503.2

**Table 4:** Number of control input transmissions under different FDI attack scenarios.

Control Strategy	Case	Number of Transmissions
	Case 1: Abrupt	15,000
Time-Triggered Control	Case 2: Incipient	20,000
	Case 3: Triangle	50,000
	Case 1: Abrupt	407
Event-Triggered Control	Case 2: Incipient	515
	Case 3: Triangle	1416

**Table 5:** The total CPU time per control step.

Control Strategy	Case	Time
	Case 1: Abrupt	0.04032
Time-Triggered Control	Case 2: Incipient	0.05187
	Case 3: Triangle	0.1352
	Case 1: Abrupt	0.001406
Event-Triggered Control	Case 2: Incipient	0.001813
	Case 3: Triangle	0.005149

## Data Availability

Dataset available on request from the authors.

## Abbreviations

The following abbreviations are used in this manuscript:

2-DOF	Two-degree-of-freedom
CBFs	Control barrier functions
DoS	Denial of service
ETC	Event-triggered control
FDI	False data injection
HRI	Human–robot interaction
ISE	Integral square error
LMI	Linear matrix inequality
LQR	Linear quadratic regulator
NN	Neural network
RMSE	Root mean square error
UUB	Uniform ultimate bounded
ZOH	Zero-order hold
